# An Energy-Efficient Redundant Transmission Control Clustering Approach for Underwater Acoustic Networks

**DOI:** 10.3390/s19194241

**Published:** 2019-09-30

**Authors:** Gulnaz Ahmed, Xi Zhao, Mian Muhammad Sadiq Fareed, Muhammad Zeeshan Fareed

**Affiliations:** 1 School of Management, Xi’an Jiaotong University, Xi’an 710049, China; gulnaz@mail.xjtu.edu.cn (G.A.); zeeshan.fareed@ist.edu.pk (M.Z.F.); 2 School of Electronic and Information Engineering, Xi’an Jiaotong University, Xi’an 710049, China; sadiqfareed@mail.xjtu.edu.cn

**Keywords:** underwater sensor network, data-similarity, control-overhead management, statistical test, sleep-awake aware

## Abstract

Underwater Acoustic Network (UAN) is an emerging technology with attractive applications. In such type of networks, the control-overhead, redundant inner-network transmissions management, and data-similarity are still very challenging. The cluster-based frameworks manage the control-overhead and redundant inner-network transmissions persuasively. However, the current clustering protocols consume a big part of their energy resources in data-similarity as these protocols periodically sense and forward the same information. In this paper, we introduce a novel two-level Redundant Transmission Control (RTC) approach that ensures the data-similarity using some statistical tests with an appropriate degree of confidence. Later, the Cluster Head (CH) and the Region Head (RH) remove the data-similarity from the original data before forwarding it to the next level. We also introduce a new spatiotemporal and dynamic CH role rotation technique which is capable to adjust the drifted field nodes because of water current movements. The beauty of the proposed model is that the RH controls the communications and redundant transmission between the CH and Mobile Sink (MS), while the CH controls the redundant inner-network transmissions and data-similarity between the cluster members. We conduct simulations to evaluate the performance of our designed framework under different criteria such as average end-to-end delay, the packet delivery ratio, and energy consumption of the network with respect to the recent schemes. The presented results reveal that the proposed model outperforms the current approaches in terms of the selected metrics.

## 1. Introduction

Underwater Acoustic Networks (UANs) are gaining popularity because of its attractive updated monitoring applications like acoustic chemical waste monitoring, target tracking and detection for military applications, assisted navigation purposes, and monitoring the health of rare marine creatures [1,2,3,4,5]. The oceanic field is very large, deep and in different shapes like square, cylindrical, and rectangular. The sensor nodes used in the underwater environment are equipped with both acoustic and radio modems. The radio or light signals are used in terrestrial communication, whereas the acoustic signals are preferred in the underwater environment for data communication because of its long transmission range. The acoustic signals are less affected by scattering, signal attenuation, and absorption loss. While on the other hand, cause more delay in data communication as these signals are moving at the speed of 1500 m/s in the underwater environment. Moreover, an acoustic channel always faces problems like limited bandwidth, low transmission speed, and higher energy consumption [6,7,8].

Different models are discussed in [7,9,10,11,12,13,14] to reduce the energy consumption of Field Nodes (FNs) in the sensor network. In these schemes, the cluster-based architecture [3,4,15,16,17,18] is proved to be more energy efficient. In UANs, a large number of battery-driven and application-specific wireless sensor nodes are deployed in the sensing field [6]. Some of the FNs are deployed closer to the Surface Sink (SS) while others are deployed away from the SS for complete coverage of the sensing field [19]. These FNs sense the information from underwater environments. Then, this sensed information is conveyed towards the SS. A FN, which is actively and constantly participating in the data relaying of other FNs will drain its battery earlier as compared to the fellow FNs [19,20,21,22,23]. The FNs deplete their batteries earlier can affect the network lifetime. Whereas, cooperative communication does not affect the SS as it is a powerful and more capable node than other FNs in term of storage, bandwidth, and battery lifetime.

Currently, Mobile Sink (MS) and Autonomous Underwater Vehicle (AUV) based schemes are designed [9,24,25,26,27] for data aggregation. In these schemes, the AUV moves in a fixed path to collect data and stops at different places to collect the data. Some other approaches [10,11,24,26,28] are designed to alleviate the energy consumption UANs. However, for large underwater sensor networks, these defined approaches do not perform persuasively due to the long AUV path as it may add latency in data collection. Furthermore, due to the limitation on the battery capacity of AUVs the relative long trajectories could not be completed. The latency problem is handled in [21,29,30] by increasing the number of AUVs. Where each AUV moves in a different path and assists with other AUVs for complete network coverage. on the other hand, this may increase the operation cost and the mobility of AUVs further increase the water current movements that severely affects the communication between neighboring AUVs and the FNs.

To overcome the issues of recent schemes [4,9,15,24,27,29], we introduce a novel region-based scheme to collect data in the harsh underwater environment through the MS, which does not involve long transmission delay due to the very long trajectory for a complete network tour. We divide the Network Sensing Field (NSF) into regions for complete coverage based on the geographical nature of the water. In each region, a Region Head (RH) is assigned to control the communication between the Cluster Head (CH) and the MS. The data from RHs is collected through the MS which can free to move inside the sensing area, but for simplicity, we make Data Collection Points (DCPs) near the RH. The MS moves from top to the bottom and stops at DCP to collect information on each tour. The contributions of our designed framework are given as:

1. We introduced a new spatiotemporal multi-cast and dynamic CH role rotation technique, which is capable of adjusting the floated FNs due to water current movements. While the drifted FNs during the transmission phase can request the new CHs for conveying their data to the SS.
2. We proposed a novel redundancy control cluster-based approach to eliminate the data-similarity through some statistical tests from the application-specific UANs. The attractiveness of the proposed model is that the RH and CH control the data-similarity between the regions and clusters, respectively. This two-level data-redundancy ensures that only the original data flow toward the final sink to save the overall network resources.
3. In AUV-based schemes [9,24,25], the optimal routes for AUV are not defined and AUV stays a long time on DCPs for data gathering which introduces latency in data-gathering and operational costs. On the other hand, in the proposed model the defined routes for the MS are optimal and it stays at DCPs for a specific time to collect information which does not cause any transmission delay.

The rest of the paper is organized as follows: Section 2 illustrates the related work and motivations. The preliminaries are defined in Section 3. We discussed the network architecture of our proposed model in detail in Section 4. Evaluation measures and simulation results for the lifetime of the network, the packet delivery rate, and the average end-to-end delay are discussed in Section 5. Finally, the conclusion is drawn in Section 6.

## 2. Related Work and Motivations

This section provides a brief overview of the existing data routing approaches designed to investigate the underwater environment [31,32]. Different techniques like probabilistic scheme [19], depth-based scheme [22], and AUV-based scheme [21,28,30] are designed to collect data in the literature of UAN. A few of them bring up the idea of a courier FN [9] to decrease the data load on-forwarder FNs. A localization-based data gathering technique is investigated in [17] to decrease the network energy load for UANs. They divided the network into different depth levels. The FNs with a higher depth level send their data packets to the FNs belong to the lower depth level in the form of a chain. FN residual energy is taken as a routing constraint for the data forwarding procedure.

In [21], the authors introduced a depth-based data gathering approach. In this model, they minimized the end-to-end data delay by reducing network throughput. Moreover, the forwarder FN selection is also based on a maximum number of neighbor FNs to avoid the data error, the data loss, and the energy hole in the network. The authors in [5] discussed a directional flood-based data gathering approach. The focus of this method is to check the quality of the links between the FNs that are taking part in the data flooding process [30]. If the quality of the link between participating FNs is poor, authors involve some other FNs to participate in the flooding process. This approach achieves reliability in data delivery at the cost of additional energy.

Redundant data consumes the network resources and deteriorate the network performance by increasing the congestion. Due to the rapid increase of internet data, many data redundancy techniques have been introduced in recent years [12,13,14]. Many current techniques provided suitable solutions to improve the network performance by removing the data redundancy in the network. It has been broadly agreed that data redundancy eradication offers great benefits in practice. Generally, the benefit of removing the data redundancy is the improved network performance in terms of higher network throughput and lower end to end delay [12,13,14]. However, the currently provided solutions are not so effective and remove a part of the original data with redundant data.

Domingo and prior in [18,20], investigated and analyzed the effect of deep and shallow wavy water on the energy consumption of the network. They engaged three types of data transmission links to observe energy consumption. These are direct transmission link, cooperative transmission link, and the cluster-based transmission link. From their experimental results, they found that the direct transmission link achieves very poor outputs in the underwater scenario. As the distance between two communicating FNs increases, data drop rate also increases due to the interference in the acoustical channel which badly affects the overall network throughput [18]. Cooperative transmission overcomes this issue and outperforms in deep wavy water. Relaying is effective to save the network energy resources, however, the cooperative communication increases the complexity level of the network. While, in the cluster-based transmission, both cooperative and direct transmission is involved. Firstly, direct transmission is utilized between member FNs and the head node to collect data. After that, the cooperative transmission link is employed for forwarding the data from the head node towards the sink. The cluster-based transmission reduces the energy consumption for member FNs [4,15,16]. However, the cluster-based transmission creates the problem of the rapid battery drainage for cluster heads, which can be solved by using the MS or the AUV. The AUVs alleviate the energy burden by forwarding the data of the cluster heads.

In AUV-based approaches [21,24,25,26], the authors consider a 3D UAN, where they keep the depth level of all the FNs same as the FNs are anchored to the floor of the ocean. By supposing such strategy they simplified the case and performed all the simulations in the 2D sensing field. At the start of the network, AUV divides the field into several clusters via Voronoi generator point strategy and transmits this information throughout the network [21,24,25]. On receiving this information, FNs decide the cluster they will join for the current round. After cluster formation and association phase, a FN is selected as a cluster head on some predefined grounds. This selected head further splits the clusters into small groups of FNs called sub-clusters [26]. However, in each round, AUV travels twice in the network for network division and data collection which increase the operational costs. Additionally, the network pays energy cost twice for the head selection in each round and then further divided into sub-clusters.

An AUV-aided underwater routing algorithm for UANs is introduced in [21]. This protocol utilized multiple numbers of AUVs with considering limited mobility in the heterogeneous acoustical channels. Authors supposed a 3D network by keeping the same depth level of all the deployed FNs. The AUV moves on a specified trajectory and stops on some fixed points to collect the data from all the FNs. Due to underwater severe conditions, deployed FNs are mobile and constantly changing their positions. These mobile FNs are considered as neighbors of the stop points. The AUV stops at the fixed points for a short time interval called the probe interval, to discover these mobile neighbors [21,24,25,26]. After recognizing these mobile neighbors, the AUV generates a transmission scheduled for them. The AUV stays for a specific time at every stop to collect the data from all the neighbor FNs. However, this probe interval of recognizing and discovering the neighbor FNs introduce the data latency.

In Hop-by-Hop Dynamic Addressing Based (H2-DAB) routing algorithm [22], the authors tried to handle the issues related to the mobility of FNs. The deployed FNs are considered at different depths, where these FNs can freely move in the horizontal directions, but the movements in vertical directions are negligible. The whole network is divided into different layers from the bottom of the ocean to the surface. The numbers of layers are depending on the transmission range of FNs and the field depth level. By taking into account the average depth of the ocean, they consider 5 to 7 layers to send data towards the surface sink. This data is forwarded from the bottom to the top in the form of a chain. However, the FNs closer to the surface sink are continuously forwarding the data of their predecessors and also sensing their fields. Because of which, theses forwarder FNs deplete their batteries earlier and this may lead to end the network lifetime [33].

**Figure 1:**
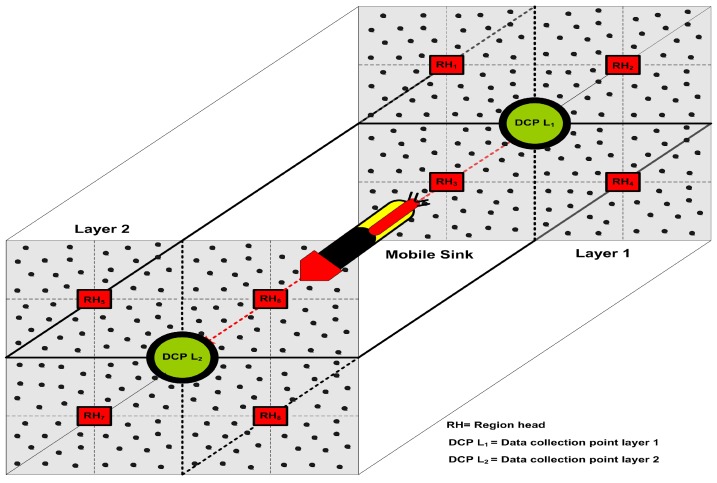
An overview of two-layer hierarchy of the proposed model.

The proposed model has some unique characteristics to deal with such problems and perform well in the harsh underwater requirements. In our designed model, data is forwarded to the SS according to the number of layers during a complete network tour. We engage the RHs to collect the frequently occurring data from the ocean bottom without causing the end-to-end delay. These deliberate RHs only collect the data from the Selected Member Nodes (SMNs) and forward the received data towards the MS after compressing it. The MS moves to a pre-defined trajectory such as from the ocean surface to the bottom and stops for a short period at each DCP to collect the data as described in Figure 1. According to the harsh underwater environment and necessities, the RTC scheme has some different features in comparison with previously designed approaches as follows:

1. In previously designed cooperative communication models [5,21,22], the FNs closer to the SS deplete their batteries earlier than expected time duration due to continuous forwarding the data of their predecessors. In our designed scheme, the FNs closer to the SS directly communicate with it. However, the remainders of the FNs forward their data through the RH and the MS.
2. The network is divided into equal size regions according to the geographical nature of the sea for load balancing and equal energy distribution among all the FNs in the network.
3. The designed model is scalable because if we add more and more layers in our model the data forwarding hierarchy remains constant and the performance of our model remains almost the same.

### Problems Statement

UANs are generally facing a series of problems like the network coverage [6,34], surface sinks positioning [35,36,37], vulnerability and data security [7,38,39], data latency [33], and energy management of deployed nodes [6]. In relation to the battery-driven underwater sensors, many AUV-based schemes [21,24,29], cluster-based schemes [15,16,17,19] and MS-based approaches [9,18] have been proposed in the literature. Even though, these developed schemes are well-organized, but not as efficient as required for the harsh underwater environment due to these subsequent reasons:

1. The previously designed schemes used the cooperative communication links to forward the data from the root FNs to the SS. However, the FNs closer to the SS always take part in relaying the data of their predecessors and also sense their NSF. This extra duty of relaying data consumes an additional amount of energy and become a root cause to deplete forwarder FN’s battery earlier as compared to the distant nodes [28,33].
2. These approaches [4,15,16,18] utilized the clustering method for forwarding the data towards the SS. However, due to poor CH selection measure and cluster size control criterion increases the burden on the large size network. That makes the network unstable and may lead to end the network lifetime earlier.
3. The multi-AUVs based approaches perform persuasively for a large network, but not perform well on few-layer networks and also increase the overall cost of the network. Sometimes, the movements of multiple AUVs increase the water current movements and badly affect the communications of neighbor AUVs and FNs.

## 3. Preliminaries

In the preliminaries section, we had given the details of the energy, and end-to-end delay models.

### 3.1. The Energy Model

To calculate the energy consumption for transmitting a *k* bit data packet at a distance *d* can be calculated through the energy model given in [2] as:

(1) $$\begin{array}{c} E_{Tx}\left(k,d\right)=\left\{\begin{array}{cc} E_{elec}k+kE_{fs}d^{2} & \;if\;d<d_{0}.\\ E_{elec}k+kE_{mp}d^{4} & \;if\;d\geq d_{0}. \end{array}\right. \end{array}$$

where $E_{elec}$ is the energy consumed per bit to run the transmitter or the receiver circuit, $E_{amp}d^{4}$ and $E_{fs}d^{2}$ symbolize the coefficient of transmit amplifier for free space model, while *d* signify the distance between sender and receiver. Furthermore, the coefficient of transmit amplifier for multi-path model is $E_{amp}d^{4}$. When a FN receives a *k* bit data packet, the energy consumed by the FN is computed using the following expression:

(2) $$\begin{array}{c} E_{Rx}\left(k\right)=kE_{elec}. \end{array}$$

In underwater, the attenuation of the signal depends on both the frequency *f* and the distance *d*. Therefore, SNR for a low bandwidth signal with the frequency and unit transmission power can be represented through ρ(d,f). An acoustic channel with a distance *d*, frequency f(KHz) and the spreading factor *F* can be computed using [3,9,40,41,42,43,44] through following expression as:

(3) $$\begin{array}{c} A\left(d,f\right)=A_{o}\left[a{\left(f\right)}^{d}\right]d^{F}. \end{array}$$

where $A_{o}$ and *A* represent the normalization constant and signal attenuation function, respectively. *F* is a spreading factor, its value depends upon the environment as:

1. F = 1 for shallow water environment.
2. F = 1.5 for practical environment.
3. F = 3 for the deep water environment.

The co-efficient of absorption a(f) can be computed by employing the Thorp formula as:

(4) $$\begin{array}{c} 10loga\left(f\right)=\left(0.11f^{2}\right)\times \frac{1}{1+f^{2}}+\left(44f^{2}\right)\times \frac{1}{4200+f}+2.75f^{2}\times 10^{-4}+0.003\;\;\;\;if\left(f>0.4\right). \end{array}$$

Here the a(f) is considered in dB/Km for the calculation purpose.

(5) $$\begin{array}{c} 10loga\left(f\right)=0.002+\left(0.11f\right)\times \frac{1}{1+f}+0.0011f\;\;\;\;if\left(f<0.4\right). \end{array}$$

Now the energy consumption between a transmitter and receiver FN for transmitting a *k* bit data packet at a distance *d* with the transmitting frequency *f* is computing using [3] as:

(6) $$\begin{array}{c} E_{Tx}\left(k,d\right)=\left\{\begin{array}{cc} E_{elec}k+ka{\left(f\right)}^{d}d^{2} & \;if\;d<d_{0}.\\ E_{elec}k+ka{\left(f\right)}^{d}d^{4} & \;if\;d\geq d_{0}. \end{array}\right. \end{array}$$

### 3.2. The End-to-End Delay Model

Signal propagation delay is calculated by using end-to-end delay model used in [42,43,44,45] as:

(7) $$\begin{array}{c} D_{p}=\frac{d}{v}. \end{array}$$

where *d* is the distance between the source and destination nodes and *v* is the speed at which signal moves in the acoustic channel given as:

(8) $$\begin{array}{c} \begin{array}{c} v=1449.05+45.7\left(\frac{t}{10}\right)-5.21{\left(\frac{t}{10}\right)}^{2}+0.23{\left(\frac{t}{10}\right)}^{3}+\left(1.333-0.126\left(\frac{t}{10}\right)+0.009{\left(\frac{t}{10}\right)}^{2}\right)\left(s-35\right)+16.3z+0.18z^{2}. \end{array} \end{array}$$

where *t* denotes the temperature in $C^{o}$, *z* denotes the water depth in materials, and *s* is water salinity given in PPT.

## 4. Redundant Transmission Control Clustering Approach

To explain RTC clustering approach, we divided its function into time steps (rounds). Then each of the round is further divided into four steps such as; (1) initialization phase, (2) cluster head selection phase, (3) data collection at cluster heads and (4) network data collection. The detail information about RTC approach is explained in the next subsections.

### 4.1. Network Architecture and Methodology

**Figure 2:**
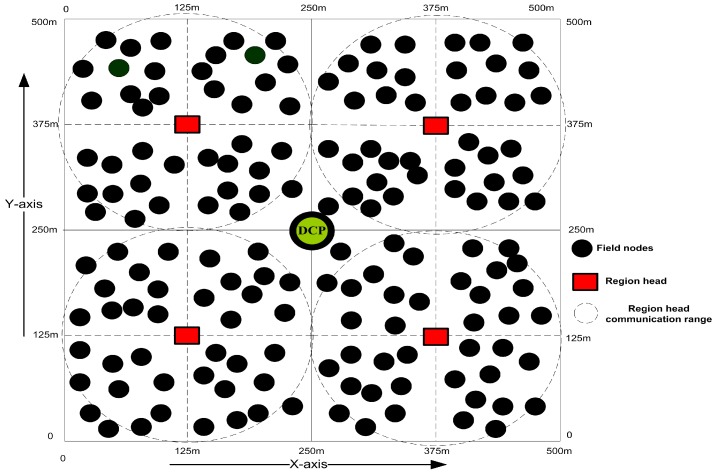
FNs and region heads deployment strategy of designed framework.

Our designed framework is application specific for the purpose of gas or oil fields monitoring, and hence sensor nodes are installed in the whole sensing field to collect the information periodically. The proposed model is very robust and has a very good delivery ratio due to the continuous field tours and good data forwarding management of the MS. It saves energy by avoiding the redundant data and repeated transmission over the link, minimizing the control packets, and sleep-awake awareness of the FNs. The SS and MS are enriched with high bandwidths and unlimited power resources. The depth of deployed FNs is considered different with the control of surface buoys [33,35,36,37]. These FNs can freely move in the horizontal direction, but the movements in the vertical direction are negligible [17]. In this way, FNs set themselves into layers from the bottom to the ocean surface. Transmission range of FNs is kept 150m by considering the average ocean depth as defined in [6,40]. In some special cases, this range can be increased, however, it is not necessary to increase this range for normal cases. We supposed an UAN which can be looked as a directional graph G=(N,L), where *N* belongs to a set of deployed nodes such as |N|=n, while *n* is the number of FNs, and *L* represents the set of links between the FNs. Furthermore, a three-dimensional rectangular cuboid area with dimensions (500 m) × (500 m) × (500 m) is taken in which the FNs are divided into four regions and each region is further divided into sub-regions as shown in Figure 2. The reason for dividing the region into cubes is that, we are not engaging all the nodes every time for sensing. So, we select some nodes from each cube for sensing and to cover the entire sensing field. The communication inside each of the region is controlled by a RH, while the CH organizes and manages the FNs entering or leaving from its cluster due to the frequent horizontal movements.

UANs communication is not similar to the terrestrial wireless communication in many aspects like low communication bandwidth due to the effect of ocean current. The designed scheme pursues the following steps:

### 4.2. Initialization Phase

In our designed model, the sensing area is partitioned into the regions and then each of the regions is partitioned into cubes. The length of the region is taken as *R* while the length of the cube is taken as *r* for further calculations. Here, we take each cube as a cluster and we adjusted *r* according to the communication range of FNs. The FNs are expressed through their location L(i,j,k) and their cluster number N(x,y,z). Whereas *x*, *y*, and *z* are computed using the following equations:

(9) $$\begin{array}{c} x=\frac{r-i\;\mathrm{mod}\;r+i}{r}. \end{array}$$

(10) $$\begin{array}{c} y=\frac{r-j\;\mathrm{mod}\;r+j}{r}. \end{array}$$

(11) $$\begin{array}{c} z=\frac{r-|k|\;\mathrm{mod}\;r+|k|}{r}. \end{array}$$

when the network configuration is complete, each RH transmits the initialization message to their corresponding clusters which contains RH location information for future correspondences. On receiving this initialization message, each of the FN computes its distance from the RH $d_{i,RH}$ and other FNs $d_{i,j}$ through this equation:

(12) $$\begin{array}{c} d_{i,j}=\sqrt{{\left(i_{1}-i_{2}\right)}^{2}+{\left(j_{1}-j_{2}\right)}^{2}+{\left(k_{1}-k_{2}\right)}^{2}}. \end{array}$$

where $\left(i_{1},i_{2}\right)$, $\left(j_{1},j_{2}\right)$ and $\left(k_{1},k_{2}\right)$ are points between FN and RH.

### 4.3. Cluster Head Selection Phase

If the basic measure of CHs selection should not be taken into account for head node selection the network lifetime may end earlier. The balanced CH selection also leads towards the less frequent re-clustering process. In our CHs selection phase, only those measures are taken into consideration that equally divide the energy and data load over the network. The CHs selection process of our model is discussed as follows:

- Each FN resets it’s timer during in the beginning of the CHs selection phase Then, FN *i* can compute it’s timer $T_{i}$ value through the following equation:
  (13) $$\begin{array}{c} T_{i}=T_{max}\times \frac{R_{EN}}{I_{EN}}\times \frac{d_{F,RH}-d_{i,RH}}{d_{F,RH}}. \end{array}$$
  (14) $$\begin{array}{c} d_{F,RH}=r\sqrt{\left(x^{2}+y^{2}+z^{2}\right)}. \end{array}$$
  where $R_{EN}$ and $I_{EN}$ are used to express the remaining and the preliminary energies of the FN, respectively. While, $d_{F,RH}$ is used to express the maximum distance between the cluster and RH. The $T_{max}$ is the maximum timer value.
- After computing the timer values, each of the FN in each of the cluster transmit a CHs advertisement message ADV_CH_SELECT at the communication range *r*. This message ADV_CH_SELECT enclose the FN’s information: FN cluster identification (x,y,z), FN location (i,j,k), distance of FN from the RH $d_{i,RH}$, $R_{EN}$ and $I_{EN}$ energies of the FN.
- Upon receiving the CHs advertisement message ADV_CH_SELECT, each of the FN analyzes the ADV_CH_SELECT and checks it’s possibility as a CH through the given expression:
  (15) $$\begin{array}{c} CH_{i}=\frac{R_{EN}}{d_{i,RH}}. \end{array}$$
  Each node has already computed its CH possibility through the above equation. So, it compares the possibility of that FN $CH_{j}$ with own $CH_{i}$. If the $CH_{j}$ is greater then $CH_{i}$ the FN update the information its table in a descending order in comparison with other ADV_CH_SELECT.
- If a FN receives ADV_CH_SELECT message after the defined time slot and the CH is not chosen yet. If so and if the $T_{i}$ of the information equals to 1, the tag $T_{i}$ of this FN is set to 0. Meanwhile, the FN who sends the ADV_CH_SELECT message is set as a CH node. Otherwise, the received message is abandoned.
- At the end of timer values, each FN is well aware with the CH possibility of all the other FNs. The FN with maximum possibility is chosen as a CH and each FN send the joint request to that FN according to their cluster table.

### 4.4. Data Collection at Cluster Heads

The CH each time selects member nodes from different directions in it’s cluster and assigns the Time Division Multiple Access (TDMA) slots to the selected member nodes. The remainders of the member nodes which do not receive TDMA slots do not take part in the sensing activity and stay in sleep-mode to save energy resources of the network. Each of the data packets received from the member nodes contains a unique packet ID, if a data packet with the same ID or containing the same information received at the CH, the CH will discards this information. On fusing the gathered information, CH checks the similarity of IDs and data packets with the previously received data packets to take any decision. If any similarity is found, then the CH will discard all those similar packets. The similarity of the received data is checked through the following tests.

#### 4.4.1. Variance Study

We perform some statistical tests to find the similarity in between the collected data. To perform these statistical tests, we assume that the variance is not substantial in all the correlated data sets. Consequently, the $S_{out}$ is calculated using the statistical tests, while $S_{out}$ is a ratio in variances which is dependent on the computed measurements. The correlated data sets are replicated each time if the value of $S_{out}$ is found lesser than the threshold $T_{DOF}$ values.

##### Assumption and Definitions for Variance Test

- Presume $N=\{N_{1},N_{2},\ldots ,N_{n}\}$ express a set of FN generating a data set $S=\{S_{1},S_{2},\ldots ,S_{n}\}$ in each slot.
- Presume $CHs=\{CH_{1},CH_{2},\ldots ,CH_{l}\}$ express a set of CHs, where l≤n and the RH gathers *n* number of data sets from the MNs in its specified region.
- Every time the collected data comprehends *T* number of measures.
- We similarly assume that the collected data sets $|S_{j}|$ are independent of number of measures the mean $\bar{X_{i}}$ though $\sigma_{n}^{2}=\sigma$.

**Definition** **1.** *When two functions having the same measures are found in a set of FN generating a data set $S=\{S_{1},S_{2},\ldots ,S_{n}\}$ can be defined as similar function and expressed as:*

(16) $$\begin{array}{c} Similar\left(s_{i},s_{j}\right)=\left\{\begin{array}{c} 1\;\;\;\;\;\;\;if\;∥s_{i},s_{j}∥\leq \delta .\\ 0\;\;\;\;\;\;\;Otherwise. \end{array}\right. \end{array}$$

*where $s_{i},s_{j}\in S$ and δ are threshold values.*

**Definition** **2.** *The measurement weight $s_{i}$ is co-occurrence of an alike function in a similar set.*

**Definition** **3.** *The cardinality of a data set $S_{n}$ is equal to the number of elements in that data set.*

**Definition** **4.** *Weighted cardinality $W_{card}$ of set $S_{n}$ is equivalent to the measure’s weight in the set $W_{card}\left(S_{n}\right)$ and the measure’s variable can be expressed [9,18] as:*

(17) $$\begin{array}{c} s_{ji}=\bar{X_{i}}+\epsilon_{ji};\;\;\;\;\;\;\;\;j=1,\ldots ,n\;and\;i=1,\ldots .,\left|S_{i}\right|. \end{array}$$

*where, $\epsilon_{ji}$ is a residual, which follows the Normal distribution $N(0,\sigma^{2})$. For the collected data sets $|S_{j}|$, we symbolize $\bar{X_{i}}$ as its mean, $\sigma_{j}^{2}$ as its variance, and $\bar{X}$ as its mean of the available data sets, respectively.*

(18) $$\begin{array}{c} \bar{X_{j}}=\frac{1}{W_{card}\left(S_{j}\right)}\sum_{k=1}^{|S_{j}|}\left(s_{jk}\times W\left(s_{jk}\right)\right). \end{array}$$

(19) $$\begin{array}{c} \sigma_{j}^{2}=\frac{1}{W_{card}\left(S_{j}\right)}\sum_{k=1}^{|S_{j}|}\left({\left(s_{jk}-\bar{X_{j}}\right)}^{2}\times W\left(s_{jk}\right)\right). \end{array}$$

(20) $$\begin{array}{c} \bar{X}=\sum_{j=1}^{n}\sum_{k=1}^{|S_{j}|}\frac{\left(s_{jk}\times W\left(s_{jk}\right)\right)}{W_{card}\left(s_{j}\right)}. \end{array}$$

*where, $s_{jk}\in S_{j}$ and $W(s_{jk})$ is measure’s weight. Since, $W_{card}\left(S_{1}\right)=\ldots =W_{card}\left(S_{i}\right)=\ldots =W_{card}\left(S_{n}\right)=T$.*

(21) $$\begin{array}{c} \bar{X_{j}}=\frac{1}{T}\sum_{k=1}^{|S_{j}|}\left(s_{jk}\times W\left(s_{jk}\right)\right). \end{array}$$

(22) $$\begin{array}{c} \sigma_{i}^{2}=\frac{1}{T}\sum_{k=1}^{|S_{j}|}\left({\left(s_{jk}-\bar{X_{j}}\right)}^{2}\times W\left(s_{jk}\right)\right). \end{array}$$

(23) $$\begin{array}{c} \bar{X}=\frac{1}{T}\sum_{j=1}^{n}\sum_{k=1}^{|S_{j}|}\left(s_{jk}\times W\left(s_{jk}\right)\right). \end{array}$$

##### Honestly Significant Difference (HSD) Test

To find the similarity in the available data sets, we perform the HSD test [19] to compute the variances and means of the available data sets. Then, we are able to choose the similarity in the available data sets and later which can completely eliminated.

(24) $$\begin{array}{c} T_{SOS}=\sum_{j=1}^{n}\sum_{k=1}^{|S_{j}|}{\left(s_{jk}\times W\left(s_{jk}\right)\right)}^{2}-\frac{{\left(\sum_{j=1}^{n}\sum_{k=1}^{|S_{j}|}s_{jk}\times W\left(s_{jk}\right)\right)}^{2}}{n\times T}. \end{array}$$

(25) $$\begin{array}{c} SOS_{between}=\frac{\sum_{j=1}^{n}\left(\sum_{k=1}^{|S_{j}|}{\left(s_{jk}\times W\left(s_{jk}\right)\right)}^{2}\right)}{T}-\frac{{\left(\sum_{j=1}^{n}\sum_{k=1}^{|S_{j}|}s_{jk}\times W\left(s_{jk}\right)\right)}^{2}}{n\times T}. \end{array}$$

(26) $$\begin{array}{c} SOS_{inside}=T_{SOS}-SOS_{between};\;\;\;DOF_{between}=n-1;\;\;\;DOF_{inside}=n\left(T-1\right). \end{array}$$

(27) $$\begin{array}{c} MOS_{between}=\frac{SOS_{between}}{DOF_{between}}. \end{array}$$

(28) $$\begin{array}{c} MOS_{inside}=\frac{SOS_{inside}}{DOF_{inside}}. \end{array}$$

(29) $$\begin{array}{c} S_{out}=\frac{MOS_{between}}{MOS_{inside}}. \end{array}$$

(30) $$\begin{array}{c} T_{DOF}=DOF\left(DOF_{between},DOF_{inside}\right). \end{array}$$

Thus, when we perform HSD test, we make sure the $S_{out}$ should lies in the probability table with an appropriate DOF while $T_{DOF}=DOF\left(DOF_{between},DOF_{inside}\right)$. This result also depends on $S_{out}$ and $T_{DOF}$:

- If $S_{out}$ > $T_{DOF}$, then in this case our assumptions are not valid because of the false rejection probabilities α, however the variance is significantly valid between the collected data.
- If $S_{out}$ < $T_{DOF}$, then in this case our assumption is valid.

#### 4.4.2. Redundancy Elimination at Cluster Heads

To eliminate the data redundancy, our designed algorithm checks the correlated data sets through the HSD test and yields a logical value. Primarily, this designed Algorithm 1 computes the value of $S_{out}$ and the threshold value $T_{DOF}$ by applying the HSD test. Finally, it yields a Boolean value if the threshold $T_{DOF}$ is greater than the variance between their measures. When the tests are completed the CH splits up and erases the similar data sets from the existing data sets. Our algorithm 1 intelligently decides which data is conveyed toward the final destination from the available data sets. Instead of forwarding all the information, only the selected information is conveyed to the RH with higher measures to increase the system efficiency and resources.

**Algorithm 1** Data-redundancy Elimination Algorithm
**Require**: set of Cluster Heads $CHs=\{CH_{1},CH_{2},\ldots .,CH_{l}\}$.**Ensure**: list of the selected sets, l.
l←∅
**for all** for each Cluster Head $CH_{i}\in CHs$ **do** consider the set $CH_{j}$ has the longest cardinality in $CH_{i}$, (i.e., $|CH_{j}|>|CH_{j*}|$; where $CH_{j*}\in CH_{i}$ ) 
$l\leftarrow l⋃CH_{j}$

**end for**
return l


### 4.5. Network Data Collection

The MS has the location information $\left(X_{m},Y_{m},Z_{m}\right)$ of each RH where *m* is the number of regions in the trajectory of the MS. After the start of the network, the MS individually visits each of the DCP, collects data from the RHs and moves toward the next DCP. On the trajectory, the number of DCPs may increase depending upon the depth levels. The RH collects data from all the CHs in a region. The CHs in each region have their unique IDs. The RH is also responsible for splitting up and erasing the similar data sets from the existing data sets. Our algorithm intelligently decides which data is conveyed toward the final destination from the available data sets. Instead of forwarding all the information, only the selected information is conveyed to the MS with higher measures to increase the system efficiency and resources.

## 5. Performance Evaluation

In performance evaluation section, we evaluate the performance of RTC by comparing it with two state-of-the-art schemes: H2-DAB [22] and AUV-PN [24]. The reason to prefer these schemes for the comparison is that these models are similar in functioning with our proposed approach. The simulation and comparison is performed using NS-3, we utilized UAN channel:: Uanchannel, CWMAC802.11DCF MAC layer protocol, NS-3 Packet Error Rate (PER) model:: UanphyperGendefault, and to recognize connection breakage underwater traversing is utilized as mentioned in [45]. The parameters selected for the simulation are given in Table 1. For a fair comparison, we take all the parameters same for all methods during the comparison. We kept the same area dimensions, the number of FNs, the network energy, and the transmission radius of nodes for all performed simulations. For the simulations, we consider the horizontal node movements like: 1 m/s to 5 m/s, while the movements in vertical directions are neglected. MS travels on a pre-defined route to collect data from all the RHs. Hello message size is considered fixed and small in accordance to one normal data packet as each Hello message consumes 0.1% of energy resources.

### 5.1. The Performance of Our Approach with Different Values of α and T

**Figure 3:**
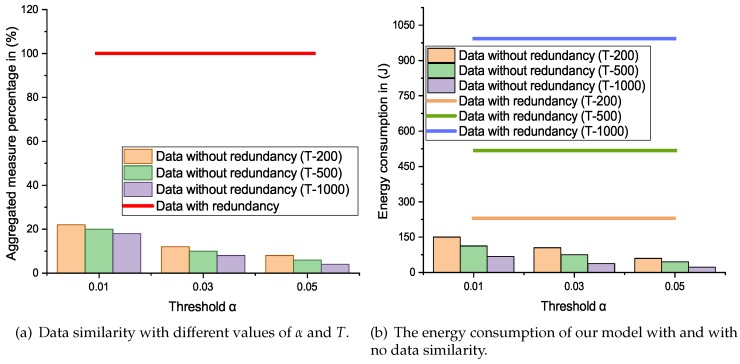
The effect of data similarity on the performance of our model.

The FNs are fixed in sensing area according to the application requirement, so most of the time the collected information is same. The Figure 3a demonstrates the data forwarded to the SS with and with no similarity. In few cases with α=0.01 and T=200 only a limited amount of without redundancy is transmitted towards the SS. From Figure 3a, we can also see that by increasing the value of α and *T* the amount of similarity in data can be further decrease to 10%. However, this may lost the original data.

The Figure 3b illustrates the amount of energy consumption with different values of α and *T*. We can also note that, the CH and the cluster member’s energy usage is associated with the number of transmissions. So, as the redundant data increases the number of transmission which affects the life of all the nodes in the network. This proposed model is designed to reduce the data redundancy with an appropriate degree of confidence and only selected data can travel toward the SS. As a result, this model is proved to be energy efficient as shown in Figure 3b.

### 5.2. Average End-to-End Delay

**Figure 4:**
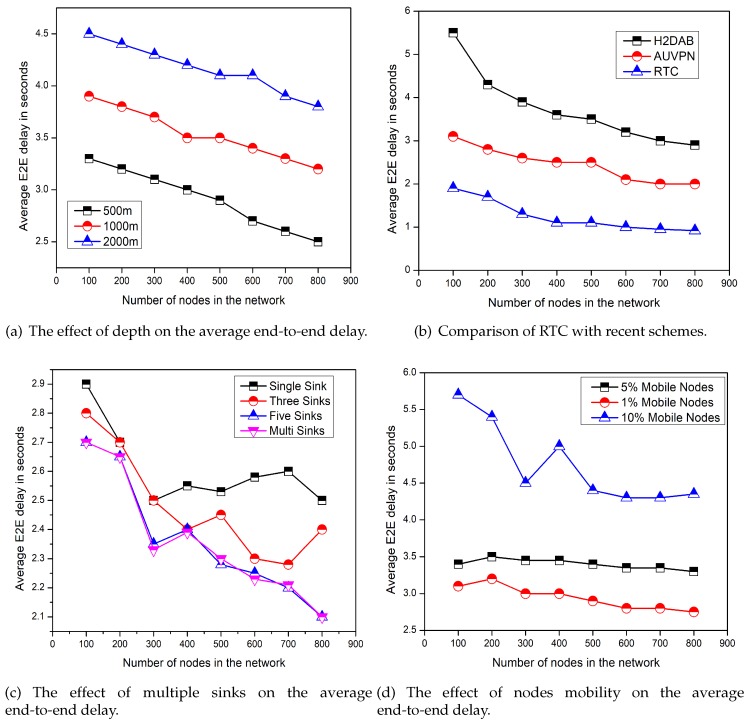
Performance of RTC by considering the average end-to-end delay.

The average end-to-end delay is related to the transmission distance and the speed of the signal in an acoustic channel. As $D_{p}=d/s$, where, *d* indicates the maximum distance between the source and destination points and *s* represents the speed of the signal which varies depending on the water depth. Figure 4 represents the end-to-end delay comparison of the proposed model with recent schemes, the effect of depth levels, the effect of node mobility, and the number of sinks. From Figure 4c,d, it is quite clear that the end-to-end delay is decreased with the help of multi-sinks and with less mobility. Because in the case of multi-sinks, data can be forwarded towards any sink without causing a little delay. Furthermore, as the FN mobility increases, there are chances that the distance between the forwarder and the sink increase, which also increase the end-to-end delay. Sometimes the connection break occurs due to the mobility of the FNs, the re-establishment of the connection also takes time and produces delay. However, there are no special effects of depth level on the average end-to-end delay as mentioned in Figure 4a. Figure 4b shows a comparison of end-to-end delay of the proposed model, AUV-PN [24], and H2-DAB [22]. The X-axis is fixed for number of nodes in the network; while on Y-axis average values for the end-to-end delay in seconds are plotted. H2-DAB has a higher end-to-end delay due to the involved number of layers for sending data to the sink. Lower layer nodes have to wait for a longer time until the courier node is reached at the closest DCP. In H2-DAB special FNs are pushed into the water with the aid of a mechanically designed element to reach the lower layer FNs to collect the data. Then, these FNs stop for a specific time interval and after that these FNs are pulled back towards the surface which also increases the end-to-end delay.

In the case of AUV-PN, the end-to-end delay is less as compared to H2-DAB [22]. On the other hand, AUV-PN has the greater end-to-end delay than RTC because in the beginning of each round AUV partitioned the sensing area into clusters. Following that the CH selection process, the AUV collects the list of PNs from the newly chosen CHs. Then AUV visits PN individually, on the arrival of AUV the PN collects data from the MNs and conveys it to AUV after fusion. The AUV sends the collected data to the surface sink after a complete network tour. This whole process takes time and increases the end-to-end delay. While in the proposed approach, the MS travels in its belonging region after a regular time intervals without staying longer at DCPs to collect the data from the associated RHs, which reduces the end-to-end delay as compared to AUV-PN.

### 5.3. Packet Delivery Ratio

**Figure 5:**
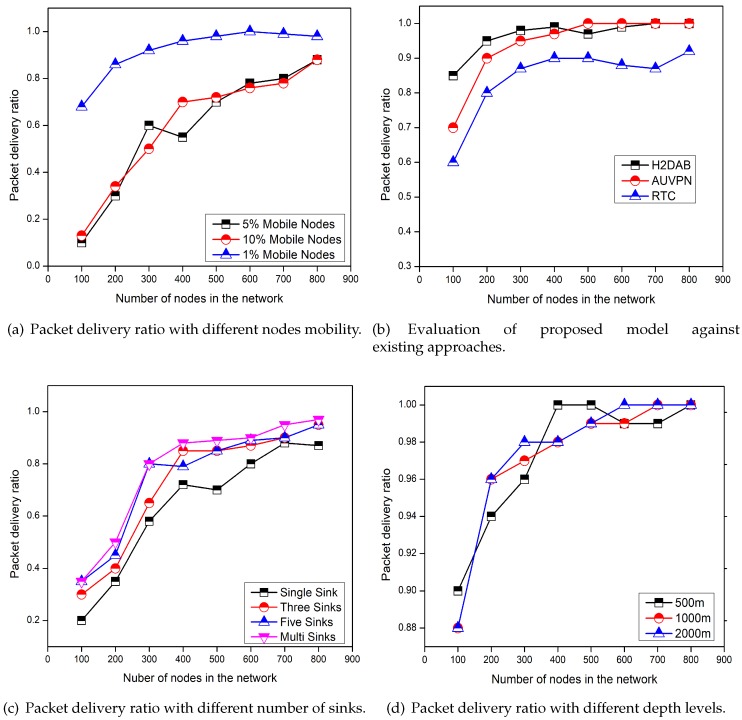
Performance analysis of proposed framework for packet delivery ratio in different scenarios.

Figure 5 represents the comparison of selected schemes for data delivery ratio and the effect of depth level, the number of sinks, and the mobility of nodes on the data delivery ratio. We can see in Figure 5a,d, the increasing node mobility and the depth level decrease the data delivery ratio. While, with the increase in the number of sinks packet delivery ratio also increases as clearly shown in Figure 5c. The reason is that, as the node mobility increases it increases the disconnections between the nodes causing the packet loss. On the other hand, the depth level has not serious effects on the packet delivery ratio. Figure 5b depicts the comparison of the packet delivery ratio among RTC, H2-DAB [22], and AUV-PN [24] schemes. The packet delivery ratio of the proposed scheme is greater than the both H2-DAB and AUV-PN. Because in RTC the less numbers of layers are involved in forwarding the data to the surface sink. Furthermore, if a FN with a data packet drifted into the neighboring sub-region due to water currents, it can easily convey its packet according to the TDMA schedule. However, a FN moved into the neighboring region will request the associated CH for forwarding the data. Then the new CH will assign a time slot and for the next round this FN will be consider as the SMNs of the new region. The H2-DAB has the lesser delivery ratio, as it delivers data towards the sink through many layers. As the more numbers of layers are involved, the probability of dropping the data packets is also increased. However, the delivery ratio of AUV-PN is lesser as compared to the RTC. Because AUV-PN has not defined any clear strategy to deal with the disconnection due to the water current movements.

### 5.4. Lifetime of the Network

**Figure 6:**
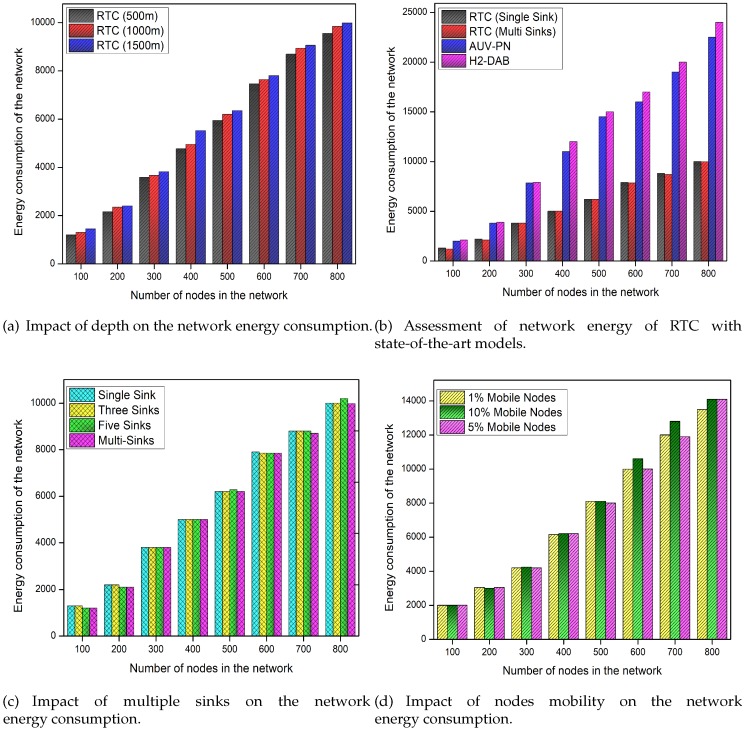
Performance evaluation of RTC based on total network energy consumption.

Figure 6 elaborates the effect of depth level, the number of sinks, and the mobility of nodes on the energy consumption of the network. We also give a comparison of energy consumption in the form of the lifetime of RTC and the state-of-the-art approaches. From Figure 6a,c, it is very much clear that varying the number of sinks and depth do not affect the lifetime of the network. The data forwarding hierarchy of the proposed scheme is very strong and involved number of sinks and depth levels cause no effect on the network lifetime. If we add more and more layers in our model, the data forwarding hierarchy remains the same and only DCPs of the MS will increase which does not affect the network lifetime. However, as the node mobility increases the network energy consumption also increases on a little scale due to the exchange of control packets as demonstrated in Figure 6d.

Figure 6b elaborates the comparison of energy consumption between RTC, H2-DAB, and AUV-PN algorithms. The given results reveal that RTC outperforms the counterpart schemes in term of energy consumption. In RTC, energy load on each FN is balanced in a distributed manner. Firstly, the FNs are forced to use the single-hop as well as the multi-hop transmission ranges to communicate with CHs depending on the situation. Secondly, RTC avoids the redundant transmissions and only selected FN can perform the sensing because nodes in the overlapping region always have the same data [19,33,35]. Thirdly, the RTC is sleep-awake aware to save the available energy of the network. The energy consumption of AUV-PN is more than RTC, as most of the network energy portion is utilized in creating the clusters. Then, in each cluster multiple head nodes are selected for data delivery, maintaining the routing table, and for correspondence with the AUV. The CH and the PN selection criteria is also very poor, which creates imbalance situation in the network. All these factors decrease the network lifetime of AUV-PN as compared to the RTC. According to depicted results, the energy consumption of H2-DAB is also higher as compared to the RTC, because it floods the control packets for finding the routes to the SS. Furthermore, it consumes a lot of energy in maintaining the routing table which leads to the shorter network lifetime.

## 6. Conclusions

In this paper, we proposed a novel redundancy control cluster-based approach to eliminate the data-similarity through some statistical tests from the application-specific UANs. We also proposed a novel spatiotemporal multi-cast and dynamic CH role rotation technique, which is capable of adjusting the floated nodes due to water current movements, while the drifted node during the transmission phase can request the new CH for conveying its data to the SS. The beauty of the proposed model is that the RH and CH control the data-similarity between the regions and clusters, respectively. In our designed scheme, the FNs closer to the SS directly communicate with the SS, while the remainders of the nodes forward their data through the RH and the MS. Furthermore, our two-level data-redundancy ensures that only the original data flow toward the final sink to save the overall network resources. We conduct a series of simulations to analyze the performance of RTC with the current schemes. The presented results reveal that the proposed model outperforms the current approaches in terms of the selected metrics. 

## Figures and Tables

**Table 1:** The values of parameters selected for the simulations.

Variable	Value
Number of FNs	100–800
Network area	500 m × 500 m × 500 m
Speed of sound	1500 m/s
Transmission range	150 m
Transmit power	50 W
Bandwidth	80 Hz
Width of layer	125 m
$E_{int}$	2000 J
$E_{elec}$	50 nJ/bit
A(f)	1.001
$d_{0}$	80–100 m
$E_{da}$	50 nJ/bit/packet
Data rate	5 Kb/s
Data packet size	64 bytes
Header size	13 bytes
Nodes mobility	1 m/s–5 m/s
Acoustic pressure of layer	101 dB
Acoustic pressure of data transmission	103 dB
Total run time	1000 s

## Abbreviations

The following abbreviations are used in this manuscript:

UANs	Underwater Acoustic Networks
RTC	Redundant Transmission Control
NSF	Network Sensing Field
AUV	Autonomous Underwater Vehicle
MS	Mobile Sink
SS	Surface Sink
FNs	Field Nodes
SMNs	Selected Member Nodes
CH	Cluster Head
DCPs	Data Collection Points
HSD	Honestly Significant Test
